# Correction: Etelcalcetide in Patients on Hemodialysis with Severe Secondary Hyperparathyroidism. Multicenter Study in “Real Life”. *J. Clin. Med.* 2019, *8*, 1066

**DOI:** 10.3390/jcm9041224

**Published:** 2020-04-24

**Authors:** Domenico Russo, Rocco Tripepi, Fabio Malberti, Biagio Di Iorio, Bernadette Scognamiglio, Luca Di Lullo, Immacolata Gaia Paduano, Giovanni Luigi Tripepi, Vincenzo Antonio Panuccio

**Affiliations:** 1 Department of Public Health, University of Naples FEDERICO II, 80131 Naples, Italy; bernadette.scognamig@alice.it (B.S.); gipaduano@libero.it (I.G.P.); 2 Institute of Clinical Physiology (IFC-CNR) Research Unit of Reggio Calabria, 89124 Reggio Calabria, Italy; rocco.tripepi@ifc.cnr.it (R.T.); gtripepi@ifc.cnr.it (G.L.T.); 3 Department of Nephrology Cremona Hospital, 26100 Cremona, Italy; f.malberti@ospedale.cremona.it; 4 Department of Nephrology AORN Cardarelli, 80131 Naples, Italy; brdiiorio@gmail.com; 5 Department of Nephrology Ospedale “Parodi Delfino” di Colleferro (Roma), Colleferro, 00034 Roma, Italy; dilulloluca69@gmail.com; 6 Nephrology, Dialysis and transplantation Unit G.O.M. “Bianchi Melacrino Morelli”, 89121 Reggio Calabria, Italy; enzopanuccio@gmail.com

The authors wish to make the following corrections to the previous publication [1] in the text, Table 1 and Table 2, and also Figure 1.

In the text on page 2, it is reported that “The following levels of serum calcium were used for the definition of hypocalcemia: < 7.0 mEq/L; ≥ 7.0 but ≤ 7.5 mEq/L; ≥ 7.5 but < 8.3 mEq/L”.

This statement needs to be corrected: “The following levels of serum calcium were used for the definition of hypocalcemia: < 7.0 mg/dL; ≥ 7.0 but ≤ 7.5 mg/dL; > 7.5 but < 8.3 mg/dL”.

We wish to correct the caption of Table 1 where serum calcium concentrations are reported as mEq/L instead of mg/dL.

The caption of the amended Table 1 is:

We wish to correct the caption of Table 2 where serum calcium concentrations are reported as mEq/L instead of mg/dL and etelcalcetide is reported as Parsabiv (trade name).

The caption of the amended Table 2 is:

We wish to correct the caption of Figure 1 where serum calcium concentrations are reported as mEq/L instead of mg/dL.

The caption of the amended Figure 1 is:

The authors apologize to the readers for any inconvenience caused by these changes. It is important to state that this correction does not affect our study’s results and involves no changes or modifications in the original data supporting our results. The original manuscript [1] will remain online on the article webpage, with reference to this Correction.

## Figures and Tables

**Figure 1:**
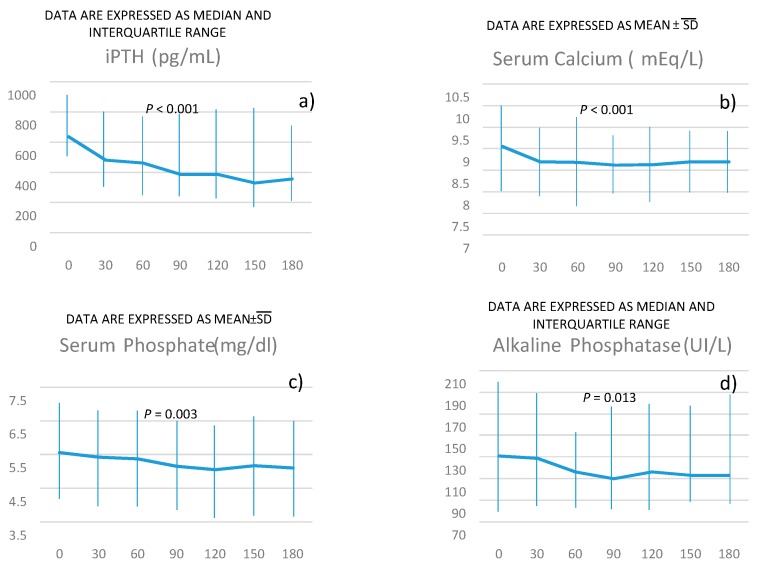
*P* for the trend of intact parathyroid hormone (iPTH) (panel **a**), serum calcium (panel **b**), serum phosphate (panel **c**), alkaline phosphatase (panel **d**) over time was obtained using linear regression models weighted for patients’ identification (see methods for more details).

**Figure 1:**
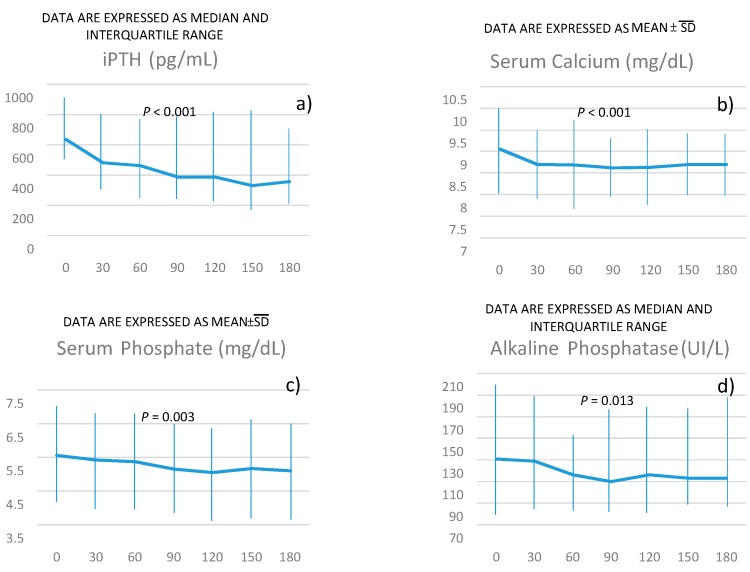
*P* for the trend of intact parathyroid hormone (iPTH) (panel **a**), serum calcium (panel **b**), serum phosphate (panel **c**), alkaline phosphatase (panel **d**) over time was obtained using linear regression models weighted for patients’ identification (see methods for more details).

**Table 1:** Patients’ Characteristics.

	Total Group (*n* = 168)	Naïve Group (*n* = 56)	Switch Group (*n* = 112)	*p* (Naïve vs Switch)
Age (years)	61 ± 14	64 ± 14	59 ± 14	0.04
Male (%)	57	52	60	0.32
Dialysis vintage (month)	58 (IQR 32–102)	35 (IQR 14–63)	69 (IQR 48–120)	<0.001
Diabetes (%)	25	31	22	0.23
Cardiovascular comorbidities (%)	73	70	75	0.53
iPTH (pg/mL)	636 (IQR 493–916)	602 (IQR 509–800)	664 (IQR 495–947)	0.67
Serum Calcium (mEq/L)	9.0 ± 1.0	9.1 ± 0.7	9.0 ± 1.1	0.60
Serum Phosphate (mg/dL)	5.6 ± 1.4	5.5 ± 1.4	5.6 ± 1.4	0.83
Alkaline Phosphate (U.I./L)	131 (IQR 83–201)	111 (IQR 74–159)	148 (IQR 88–221)	0.02
Hb (gr/dL)	11.1 ± 1.4	11.0 ± 1.2	11.1 ± 1.4	0.58
ESA treatment (%)	87	88	87	0.97
Phosphate binders therapy (%)	96	93	97	0.17
Calcium containing binders (%)	18	25	14	0.09
Vitamin D therapy (%)	75	83	71	0.09
Native Vitamin D therapy (%)	5	4	6	0.59
Previous cinacalcet treatment (%)	67	0	100	N/A

IQR, interquartile range; iPTH, intact parathyroid hormone; Hb, hemoglobin; ESA, erythropoietin-stimulating agent.

**Table 1:** Patients’ Characteristics.

	Total Group (*n* = 168)	Naïve Group (*n* = 56)	Switch Group (*n* = 112)	*p* (Naïve vs Switch)
Age (years)	61 ± 14	64 ± 14	59 ± 14	0.04
Male (%)	57	52	60	0.32
Dialysis vintage (month)	58 (IQR 32–102)	35 (IQR 14–63)	69 (IQR 48–120)	<0.001
Diabetes (%)	25	31	22	0.23
Cardiovascular comorbidities (%)	73	70	75	0.53
iPTH (pg/mL)	636 (IQR 493–916)	602 (IQR 509–800)	664 (IQR 495–947)	0.67
Serum Calcium (mg/dL)	9.0 ± 1.0	9.1 ± 0.7	9.0 ± 1.1	0.60
Serum Phosphate (mg/dL)	5.6 ± 1.4	5.5 ± 1.4	5.6 ± 1.4	0.83
Alkaline Phosphate (U.I./L)	131 (IQR 83–201)	111 (IQR 74–159)	148 (IQR 88–221)	0.02
Hb (gr/dL)	11.1 ± 1.4	11.0 ± 1.2	11.1 ± 1.4	0.58
ESA treatment (%)	87	88	87	0.97
Phosphate binders therapy (%)	96	93	97	0.17
Calcium containing binders (%)	18	25	14	0.09
Vitamin D therapy (%)	75	83	71	0.09
Native Vitamin D therapy (%)	5	4	6	0.59
Previous cinacalcet treatment (%)	67	0	100	N/A

IQR, interquartile range; iPTH, intact parathyroid hormone; Hb, hemoglobin; ESA, erythropoietin-stimulating agent.

**Table 2:** Cases of hypocalcemia.

Days after Parsabiv	<7.0 mEq/L	≥7.0 or <7.5 mEq/L	≥7.5 or <8.3 mEq/L
30	3/168 (1.8%)	0	25/168 (14.9%))
60	1/129 (0.8%)	7/129 (5.4%)	28/129 (21.7%)
90	2/111 (1.8%)	2/111 (1.8%)	27/111 (24.3%)
120	1/80 (1.3%)	1/80 (1.3%)	21/80 (26.2%)
150	1/61 (1.6%)	6/61 (9.8%)	11/61 (18.0%)
180	0	1/44 (2.3%)	11/44 (25.0%)
210	0	1/51 (2.0%)	15/51 (29.4%)

**Table 2:** Cases of hypocalcemia.

Days after Etelcalcetide	<7.0 mg/dL	≥7.0 or ≤7.5 mg/dL	>7.5 or <8.3 mg/dL
30	3/168 (1.8%)	0	25/168 (14.9%))
60	1/129 (0.8%)	7/129 (5.4%)	28/129 (21.7%)
90	2/111 (1.8%)	2/111 (1.8%)	27/111 (24.3%)
120	1/80 (1.3%)	1/80 (1.3%)	21/80 (26.2%)
150	1/61 (1.6%)	6/61 (9.8%)	11/61 (18.0%)
180	0	1/44 (2.3%)	11/44 (25.0%)
210	0	1/51 (2.0%)	15/51 (29.4%)

## References

[1] Russo D., Tripepi R., Malberti F., di Iorio B., Scognamiglio B., di Lullo L., Paduano I.G., Tripepi G.L., Panuccio V.A. (2019). Etelcalcetide in Patients on Hemodialysis with Severe Secondary Hyperparathyroidism. Multicenter Study in “Real Life”. J. Clin. Med..

